# Incorporating Consumers’ Needs in Nutrition Apps to Promote and Maintain Use: Mixed Methods Study

**DOI:** 10.2196/39515

**Published:** 2023-06-20

**Authors:** Sandra van der Haar, Ireen Raaijmakers, Muriel C D Verain, Saskia Meijboom

**Affiliations:** 1 Wageningen Food & Biobased Research Wageningen University & Research Wageningen Netherlands; 2 Wageningen Economic Research Wageningen University & Research Wageningen Netherlands

**Keywords:** mobile health, mHealth, mHealth apps, nutrition apps, diet apps, consumer needs, app, use, nutrition, tool, consumer, eating, habit, users, dietary behavior, reliable, food, database, time, developers, mobile phone

## Abstract

**Background:**

Nutrition apps seem to be promising tools for supporting consumers toward healthier eating habits. There is a wide variety of nutrition apps available; however, users often discontinue app use at an early stage before a permanent change in dietary behavior can be achieved.

**Objective:**

The main objective of this study was to identify, from both a user and nonuser perspective, which functionalities should be included in nutrition apps to increase intentions to start and maintain use of these apps. A secondary objective was to gain insight into reasons to quit using nutrition apps at an early stage.

**Methods:**

This study used a mixed methods approach and included a qualitative and a quantitative study. The qualitative study (n=40) consisted of a home-use test with 6 commercially available nutrition apps, followed by 6 focus group discussions (FGDs) to investigate user experiences. The quantitative study was a large-scale survey (n=1420), which was performed in a representative sample of the Dutch population to quantify the FGDs’ results. In the survey, several app functionalities were rated on 7-point Likert scales ranging from 1 (very unimportant) to 7 (very important).

**Results:**

A total of 3 different phases of app use, subdivided into 10 user-centric app aspects and 46 associated app functionalities, were identified as relevant nutrition app elements in the FGDs. Relevance was confirmed in the survey, as all user-centric aspects and almost all app functionalities were rated as important to include in a nutrition app. In the starting phase, a clear introduction (mean 5.45, SD 1.32), purpose (mean 5.40, SD 1.40), and flexible food tracking options (mean 5.33, SD 1.45) were the most important functionalities. In the use phase, a complete and reliable food product database (mean 5.58, SD 1.41), easy navigation (mean 5.56, SD 1.36), and limited advertisements (mean 5.53, SD 1.51) were the most important functionalities. In the end phase, the possibility of setting realistic goals (mean 5.23, SD 1.44), new personal goals (mean 5.13, SD 1.45), and continuously offering new information (mean 4.88, SD 1.44) were the most important functionalities. No large differences between users, former users, and nonusers were found. The main reason for quitting a nutrition app in the survey was the high time investment (14/38, 37%). This was also identified as a barrier in the FGDs.

**Conclusions:**

Nutrition apps should be supportive in all 3 phases of use (start, use, and end) to increase consumers’ intentions to start and maintain the use of these apps and achieve a change in dietary behavior. Each phase includes several key app functionalities that require specific attention from app developers. High time investment is an important reason to quit nutrition app use at an early stage.

## Introduction

### Background

Healthy dietary habits play a crucial role in preventing obesity and noncommunicable diseases such as cardiovascular diseases, cancer, and diabetes mellitus [1,2]. Despite its importance, the dietary intake of many consumers in the Western world is suboptimal (eg, excessive intake of sodium and insufficient intake of whole grains and fruits), leading to a high global disease burden [3]. Therefore, there is an urgent need for interventions and tools that stimulate and support a healthy eating pattern. Increased access to smartphones, tablets, and wearables has caused an increase in the popularity of mobile health (mHealth) apps. Currently, over 350,000 mHealth apps are available in the health and fitness category in various app stores worldwide [4]. There is evidence that mHealth apps are effective or likely to be effective in stimulating healthier behaviors, such as increasing physical activity [5,6], reducing sedentary behavior [5], improving dietary habits and intake [5-7], and losing weight [8,9]. Nutrition apps are a part of the mHealth category and specifically focus on tracking food intake and providing dietary advice. Most of these apps function as food diaries in which users log their daily food intake, either by a text search or a barcode scanner [10]. The app subsequently gives the user an overview of daily amounts of calories and other nutrients consumed and provides them with dietary advice. Nutrition apps are considered promising tools for supporting consumers in the transition toward a healthier diet. A study by Wang et al [11] showed that consumers perceive the effectiveness of nutrition apps as rather high. In their study, they included both nutrition and physical activity apps. The use of both types of apps influenced action, consciousness, self-education about nutrition and physical activity, and social life (eg, by sharing dietary experiences in web-based social networks). Furthermore, it facilitated maintaining a healthy diet and exercising more [11].

An important question is which elements are important for nutrition apps to be effective in achieving healthy eating behaviors. Several studies examined the application of behavior change techniques (BCTs) in mHealth apps. BCTs are components of behavior change interventions that can be used alone or in combination with other BCTs, such as goal setting, self-monitoring, and feedback [12]. The inclusion of BCTs seems beneficial to the quality of mHealth apps [13-15] and might in turn influence consumer behavior [16]. This is in line with previous research showing that health behavior interventions are more effective when they integrate such techniques [17,18]. According to several studies, the extent to which BCTs are incorporated into mHealth apps is still insufficient at this point [15,16,19] or is only sufficient in paid versions of the app [20].

Besides the incorporation of science-based components in mHealth interventions, such as BCTs, it is of great importance to focus on the issue of implementation. A key factor in mHealth implementation is the willingness and capability of users to successfully engage with a tool or app [21]. This is an important precondition for both the *efficacy,* the capacity of a given mHealth intervention in a controlled setting, and the *effectiveness,* to have a meaningful effect on users in real life [22]. The user-friendliness of mHealth app use of advertisements, price, and protection of personal data and privacy are examples of aspects that potentially influence the implementation and acceptance of these tools [23-26].

Nowadays, a wide variety of nutrition apps are available; however, only a small group of Dutch consumers (11%) make use of such apps [27]. Users often prematurely discontinue use before a change in dietary behavior can be achieved, indicating possible issues with the implementation of these apps. This was shown by Helander et al [28] in a retrospective study. They concluded that most people who tried out a free mobile app for dietary self-monitoring did not continue using it actively [28]. There might be various barriers for consumers to use a nutrition app or to quit its use at an early stage. A recent systematic literature review by König et al [29] identified 328 barriers and facilitators for nutrition app use. The usability of the app was the most frequently identified barrier in this review [29]. The user burden of nutrition apps is rather high because tracking all eating and drinking moments is a time-consuming activity. Furthermore, there can be several issues with the food tracking feature and the underlying food database in nutrition apps. A study by Ziesemer et al [30] demonstrated that usability issues related to tracking food intake might impact the willingness to record eating events. In addition, Vasiloglou et al [25] showed that consumers would not select nutrition apps that have issues related to their food and nutrient databases, such as an incomplete product list or incorrect estimations of nutrients.

### Objectives

To summarize, several factors could contribute to consumers’ intentions to use nutrition apps and the early discontinuation of these apps. Therefore, the main objective of this study was to identify, from both a user and nonuser perspective, which functionalities should be included in nutrition apps to increase intentions to start and maintain use of these apps. A secondary objective was to gain insight into reasons to quit using nutrition apps at an early stage.

## Methods

### Study Design

The study followed a mixed methods approach and consisted of 2 parts: a qualitative and a quantitative study. The qualitative study consisted of a home-use test with commercially available nutrition apps, followed by focus group discussions (FGDs). The results of the FGDs served as a basis for the quantitative part, a survey that aimed to quantify these results in a large, representative sample of the Dutch population.

### Ethics Approval and Informed Consent

All participants provided written informed consent for participation in the study. In addition, participants in the qualitative study provided consent for audio recordings of the discussions. Ethics approval for the study was obtained from the Social Ethics Committee of Wageningen University & Research in the Netherlands.

### Qualitative Study

#### Recruitment and Study Procedures

Participants were recruited from the Wageningen Food and Biobased Research consumer database. This database consists of consumers who are interested in participating in nutrition and health research and live in the Wageningen region. An email invitation to participate in this study was sent to a subsample of the panel (750 consumers) using random selection. Inclusion criteria were age between 18 and 60 years and familiarity with smartphone and app use. A total of 62 participants signed up for the study, of which 48 (77%) were invited to participate. There were 3 dropouts during the home-use test and 5 no-shows at the FGDs; therefore, a total of 40 participants completed the study.

Study participants first took part in a home-use test. In total, 6 commercially available, free nutrition apps in which food intake could be tracked were selected for this test: MyFitnessPal, FatSecret, Lifesum, Mijn Eetmeter, FoodProfiler, and SamenGezond. The apps were selected based on a short literature search to identify the prerequisites of successful mHealth apps and their differences in BCTs (goal setting, goal tracking, monitoring, feedback, rewards, social support, identification, game elements, and personalization) and other app functionalities (prompts, synchronization with other apps and devices, and costs). The apps were used as a tool to start the conversation on user experiences and critical app elements; the aim was not to test or rate these specific apps. Participants were asked to download and use 1 of these 6 apps for a period of 3 weeks. Some participants had previous experience with 1 of the apps used in the test. In that case, they were assigned to a different app that was new to them because we also wanted to include their first experience of using the app. After 3 weeks of app use, 6 semistructured FGDs (1 per included app) of 1.5 hours were organized at the Wageningen University & Research campus. The discussions were led by a professional focus group moderator. A focus group guide was designed and used during the discussion to ensure the comparability of the 6 FGDs. The main objective of the discussions was to identify, from a user perspective, what were the critical elements for successfully monitoring and supporting healthy eating behaviors. Another objective was to explore possible reasons to quit using the specific app. In each session, 6 to 8 consumers participated, who all tested the same nutrition app before their session. The 3 main topics addressed in the FGDs were general smartphone use, use of health and nutrition apps, and user experiences with the tested nutrition app. The latter topic was discussed extensively. Participants were asked to describe their positive and negative experiences and how often they had used the app. Subsequently, the app functionalities were discussed and evaluated regarding their usefulness. The functionalities differed per app, but in all focus groups, both predefined intervention components of the app (BCTs such as goal setting, feedback, reward, social support, and knowledge) and the implementation of these components (eg, use aspects such as reminders, chat, synchronization with other devices, and gamification element) were discussed. Audio recordings were conducted, and minutes were recorded for each session. Upon completion of the study, the participants received an incentive of €50 (conversion rate at the time of the study: €1=US $1.12) for their time investment.

#### Analyses

**Figure 1:**
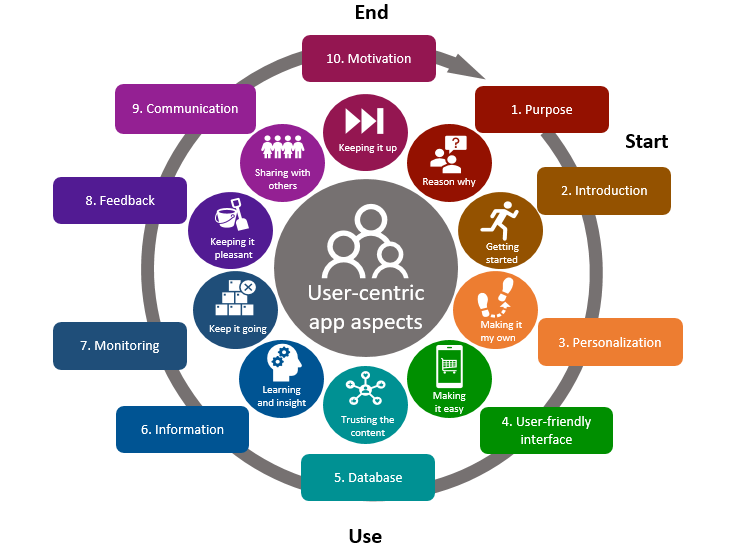
Overview of the 3 phases of nutrition app use subdivided into 10 “user-centric app aspects.”.

As a first step, the minutes and recordings of the discussions were analyzed by creating a descriptive matrix per question to identify common themes. Through this matrix, the reasons for discontinuation were identified. The model shown in Figure 1 was built using a bottom-up approach. On the basis of the app evaluation, user quotes from the participants were translated into the app properties. For example, the user quote “I would use the products that were placed under the dinner category at other moments” was translated into the key app property: “logical product categories.” Thus, all user quotes related to the tested app were translated, resulting in a list of 46 properties (Table S1 in Multimedia Appendix 1). These app properties were then assigned to 10 different categories (also called “user-centric app aspects”; Table S1 in Multimedia Appendix 1). These categories were partly derived from the predefined list of BCTs and other app properties in the focus group guide (eg, “Monitoring”) and partly from topics participants came up with themselves in the discussion (eg, “Database”). In this manner, the model with 10 different user-centric aspects and 46 app properties was built. In the analysis, it was not about the frequency of the quotes but about their unicity, as the aim was to generate a complete overview of user experiences.

The 3 different phases of app use were identified as the final step. Some of the user-centric app aspects particularly occurred when the user installed the app and during the first (few) time(s) of use, the *start phase*. Some aspects typically occurred during daily use, the *use* *phase*. Most participants reached a point where the app would be abandoned or used permanently, the *end phase*. This was related to a combination of aspects in the 2 earlier phases and the continuous engagement of the user. The phases were placed in the outer ring of the model.

The analyses of all 6 FGDs were performed by the same professional focus group moderator who had led the discussions. At least 1 of the researchers was present at each focus group session, and afterward, the minutes and analyses of the results were reviewed by the researcher.

### Quantitative Study

#### Sampling and Study Procedures

A web-based survey was conducted in a representative sample of the Dutch adult population (>18 years), familiar with the use of smartphone apps and either with or without experience regarding the use of nutrition apps. The survey was administered by a professional market research agency (MSI-ACI Europe Ltd). Quota sampling was applied to obtain a representative sample for age, gender, level of education, region, and income. Participants were approached by email to fill out a web-based self-administered survey and received an incentive in the form of credits for a personal saving system.

The survey included questions on nutrition app use, reasons for using and for not using nutrition apps, and the importance of 46 nutrition app functionalities that resulted from the FGDs. In total, 1500 participants completed the survey. During data cleaning, 80 participants were removed from the analyses because they showed no dispersion in their answers on the importance of app functionalities, suggesting that they did not fill out this part of the survey in earnest.

#### Measures

##### Nutrition App Use

Respondents were asked to indicate whether they make use of nutrition apps or made use of nutrition apps in the past. On the basis of their responses, they were categorized into former users, previous users, and nonusers. Subsequently, they were asked to indicate their reasons for (not) using nutrition apps, for example, “Because I want to lose weight” or “I never heard of nutrition apps.” Former users were asked what was their reason or were their reasons for quitting the use of the app, for example, “It cost me too much time.” Respondents could select multiple options from a predefined list of reasons or fill in an open answer.

##### Nutrition App Functionalities

The 46 nutrition app functionalities that resulted from the FGDs (Table S1 in Multimedia Appendix 1) were included in the survey. For each app functionality (eg, “A quick and easy entry of food products”), participants were asked how important they thought this functionality was to include in a nutrition app. The items were randomized into 4 subsets and assessed on 7-point Likert scales ranging from 1 (very unimportant) to 7 (very important).

##### Demographics

Age, gender, and education level were included in the survey to analyze the sample on sociodemographic characteristics.

#### Statistical Analysis

Categorical variables were displayed as frequencies and percentages, and numeric variables were displayed as mean (SD). Respondents were categorized into *users* and *nonusers* according to their nutrition app use behavior. The group of users consisted of *current users* and *former users*. The number of participants in each group and the percentage of the total study population were calculated. The mean (SD) scores were calculated for the importance ratings of each nutrition app functionality. The top 3 most important app functionalities per phase were created based on these mean ratings. The 10 user-centric app aspects included multiple app functionalities. Per user-centric app aspect, mean (SD) scores were calculated, combining all app functionalities within the user-centric app aspects (refer to Table S1 in Multimedia Appendix 1 for an overview). One-way ANOVA was used to compare the means of the 3 different groups (nonusers, current users, and former users). The Brown-Forsythe ANOVA with Games-Howell post hoc tests was applied to account for unequal samples and variances. Statistical significance was set at *P*<.001 for all analyses. Statistical analyses were performed using SPSS software (version 25.0; IBM Corp).

## Results

### Qualitative Study

#### Phases of App Use and User-Centric App Aspects

A total of 40 participants (8 male individuals and 32 female individuals, with a mean age of 40.9, SD 14.1 years) participated in the FGDs. None of the participants had a low education level, 15% (6/40) of the participants had a medium education level, and 85% (34/40) of the participants had a high education level. The FGDs revealed that users go through 3 different phases when using a nutrition app: start, use, and end. Each phase includes a range of key aspects or categories. In total, 10 such “user-centric app aspects” were identified (Figure 1). Each of these aspects includes a total of 46 different key app functionalities (refer to Table S1 in Multimedia Appendix 1 for a complete overview).

#### App Functionalities

In the *starting phase,* the purpose (user-centric app aspect 1, Figure 1) of the app should be clear immediately, and a clear introduction (user-centric app aspect 2, Figure 1) to the different functionalities of the app should be present (user: “A tutorial that you can view optionally would be helpful”). The next aspect is personalization (user-centric app aspect 3, Figure 1) of the app by entering personal data and goals (user: “When you create your own list of recipes, you only have to fill it in once, which is convenient”). In addition, certain other app functionalities must be adjustable according to personal wishes, such as how often notifications appear. During the *use phase*, users go through several aspects of the app, either sequentially or simultaneously. First, user-friendliness (user-centric app aspect 4, Figure 1) is of great importance in this phase, especially a quick and easy daily food intake entry (user: “Efficient entry is important. I am very impatient”). Moreover, the product database (user-centric app aspect 5, Figure 1) in the app must be of good quality and not be contaminated with duplicate products or incorrect nutritional information (user: “There is no added value of having so many similar products in the list”). Furthermore, the information (user-centric app aspect 6, Figure 1) or advice provided by the app should educate the user (user: “I didn’t know almonds contained so many calories.”). In addition, the user must gain sufficient insight into their own dietary behavior and progress by monitoring (user-centric app aspect 7, Figure 1) functions in the app (user: “I adjusted my behaviour based on the daily overview of what I ate”). Visualizing progress toward achieving personal goals can be helpful. Users prefer to receive positive feedback (user: “I liked the encouraging tone of voice of the feedback messages”; user-centric app aspect 8, Figure 1), a variety of different feedback messages, or a game element as a way to provide feedback. In addition, a reward system where, for example, credits can be earned by keeping up with entering food intake daily has a stimulating effect on continued use of the app. Furthermore, some appreciate the possibility of communication (user-centric app aspect 9, Figure 1) with other users (via a community, forum, or social media) or with a web-based coach (user: “I entered a goal to eat more vegetable and the coach would give tips”). In the *end phase,* continuous engagement (user-centric app aspect 10, Figure 1) of the user is particularly important (user: “It took forever to fill the app. That really demotivated me”). In addition, the app should continuously offer new and relevant information to keep users interested and engaged.

#### Reasons to Quit Use

##### Entering Food Intake and the Database

Most participants in the home-use test discontinued the use of the nutrition app prematurely. Although some participants did mention a few advantages, such as an increased awareness of personal food intake and dietary habits, most participants experienced too many disadvantages. The poor user-friendliness of the app made the time investment too high for most users. This was mainly linked to the time-consuming task of registering daily food intake. If entering food products could not be achieved quickly and easily, this was a big barrier (user: “I had to type in the same food products over and over again”). In some apps, users could add their own food products to the food product database. This caused contamination of the database, with too many details and options (user: “Full-fat yoghurt had so many entries, with all different amounts of calories.”). In contrast, sometimes the database was too limited or incomplete for specific product categories (user: “There were five different options for chocolate milk, but only one type of cheese”). These issues sometimes caused the user to not trust the content of the database (user: “The app showed very different calorie amounts for different apples, it made me question the reliability”).

##### Feedback and Advice

In some cases, the way feedback and advice were framed caused the user’s continuous engagement to decline. Some apps used a patronizing tone of voice (user: “I want to be aware of what I eat, but don’t be judged through the advice given.”) or were too rigid in the feedback provided (user: “One calorie too much and you are in the red zone. That’s too abrupt”). For an app to remain relevant and triggering for a longer period, it needs to constantly offer new information to the user. Some users indicated that the advice or tips were too repetitive or already known (user: “The app tells me nuts are healthy. I know that already, so I deliberately entered that I ate nuts just to get rid of this notification.”). Some participants indicated that continued use of a nutrition app would be unlikely for them. In the beginning, there can be a steep learning curve because, as a new user, you become aware of your dietary pattern and learn about the macronutrient composition of products and healthier product alternatives. In this phase, a nutrition app can have a lot to offer, and the time and effort to track food intake daily pays off in insights, support, and suggestions to improve the diet. However, after a certain amount of time, when certain behaviors might have been adapted and the learning curve flattens, the necessity and relevance of a nutrition app diminishes (user: “You have to use these type of apps to teach yourself good behaviour. And then you have to put them away”). Still, some users think it is a good idea if the app would notify or email them after a couple of months to remind them of their goals and increase their awareness.

### Quantitative Study

#### Nutrition App Functionalities

A total of 1420 participants were included in the final analyses. The sample was nearly equally distributed in terms of gender, with 49% (696/1420) of male individuals and a mean age of 45.7 (SD 16.5) years, with an age range of 18 to 79 years. The majority had a middle (626/1420, 44%) or high (579/1420, 41%) level of education. The sample was representative for the Dutch population regarding age and gender. Respondents with a low education level were somewhat underrepresented, and respondents with a high education level were overrepresented. Almost all app functionalities (43 out of 46) were rated with a mean score above the neutral score of 4. The possibility of linking the nutrition app with social media (mean 3.8, SD 1.9), a gamification element in the app (mean 3.8, SD 1.9), and the possibility of being in touch with other users through social media platforms (mean 3.5, SD 1.8) were the only functionalities rated with a mean score <4. The complete list of mean ratings per app functionality can be obtained from Table S2 in Multimedia Appendix 1. Table 1 shows the mean importance ratings for the 10 user-centric app aspects, which all include multiple app functionalities (refer to Table S1 in Multimedia Appendix 1 for the associated functionalities). The user-centric app aspects of purpose, introduction, user-friendliness, database, and information received the highest mean ratings (all ≥5).

In Table 2, the top 3 most important app functionalities per use phase and the user-centric app aspect to which they belong are displayed (refer to Table S2 in Multimedia Appendix 1 for the full list of app functionality ratings). In the starting phase, the top 3 app functionalities include a clear introduction and the purpose of the app. Furthermore, flexibility in food tracking is important. In the use phase, app functionalities relating to the product database and the user-friendliness of the app were rated as particularly important. In the end phase, the most important app functionalities were the possibility of setting achievable goals, setting new personal goals, and offering new information to the user continuously.

Table 3 shows the mean importance ratings for the 10 user-centric app aspects per user group (*current users,* *former users,* and *nonusers*). Significant differences between the groups were found in the following app aspects: personalization (*P*<.001), user-friendliness (*P*<.001), database (*P*<.001), and monitoring (*P*<.001). Regarding personalization, user-friendliness, and monitoring, *current users* gave significantly higher ratings than *nonusers*. Regarding the database, *former users* gave significantly higher ratings than *nonusers*. Although the differences between these groups were significant, the mean ratings for all 3 user groups were still very close to each other (ranging from a 0.2 to 0.7 difference on a 7-point Likert scale).

**Table 1:** Mean importance rating per user-centric app aspect (n=1420).

Phase and user-centric app aspect^a^		Values, mean (SD)
**Start**		
	Purpose	5.1 (1.2)
	Introduction	5.2 (1.2)
**Use**		
	Personalization	4.9 (1.1)
	User-friendliness	5.2 (1.1)
	Database	5.4 (1.2)
	Information	5.0 (1.2)
	Monitoring	4.7 (1.2)
	Feedback	4.4 (1.3)
	Communication	4.4 (1.2)
**End**		
	Continuous engagement	4.9 (1.2)

^a^Measured on a 7-point Likert scale (1=very unimportant and 7=very important).

**Table 2:** Top 3 most important nutrition app functionalities per use phase and the corresponding user-centric app aspect (n=1420).

Phase and app functionality^a^			Values, mean (SD)		User-centric app aspect
**Start**					
	Immediately clear how app should be used	5.45 (1.32)		Introduction	
	The app has a clear purpose	5.40 (1.40)		Purpose	
	Possibility to track food intake at own time	5.33 (1.45)		Personalization	
**Use**					
	Complete and reliable product database	5.58 (1.41)		Database	
	Easy navigation through the app	5.56 (1.36)		User-friendliness	
	Limited advertisements in free version	5.53 (1.51)		User-friendliness	
**End**					
	Possibility to set realistic and achievable goals	5.23 (1.44)		Continuous engagement	
	Possibility to set new personal goals	5.13 (1.45)		Continuous engagement	
	New and relevant information is continuously offered	4.88 (1.44)		Continuous engagement	

^a^Measured on a 7-point Likert scale (1=very unimportant and 7=very important).

**Table 3:** Mean importance rating per user-centric app aspect, comparing 3 different user groups (current, former, and nonusers).

Phase and user-centric app aspect			Current users (n=276), mean (SD)		Former users (n=38), mean (SD)		Nonusers (n=1106), mean (SD)		*P* value
**Start**									
	Purpose	5.35 (0.90)		5.22 (0.84)		5.09 (1.26)		.004	
	Introduction	5.31 (1.00)		5.11 (0.97)		5.21 (1.30)		.40	
**Use**									
	Personalization	5.21 (0.81)^a^		5.11 (0.67)^a,b^		4.77 (1.16)^b^		<.001	
	User-friendliness	5.34 (0.87)^a^		5.57 (0.69)^a,b^		5.10 (1.10)^b^		<.001	
	Database	5.57 (0.95)^a,b^		5.94 (0.76)^a^		5.34 (1.22)^b^		<.001	
	Information	5.21 (1.00)		5.16 (0.98)		4.91 (1.25)		.001	
	Monitoring	5.02 (1.01)^a^		4.66 (1.14)^a,b^		4.68 (1.28)^b^		<.001	
	Feedback	4.65 (1.25)		4.33 (1.32)		4.38 (1.26)		.005	
	Communication	4.59 (1.24)		4.16 (1.20)		4.35 (1.23)		.007	
**End**									
	Continuous engagement	5.17 (0.98)		5.17 (0.90)		4.88 (1.26)		.001	

^a,b^Cells with the same letters indicate no significant difference following the post hoc analysis.

#### Nutrition App Use and Reasons to Quit

Approximately one-fifth (314/1420, 22.1%) of the respondents had experience using a nutrition app, either in the past or at the time of filling out the survey. The majority (1106/1420, 77.9%) never made use of a nutrition app. Within the group of users, a distinction could be made between *current users* (276/1420, 19.4%) and *former users* (38/1420, 2.7%).

Table 4 shows the most important reasons to make use of a nutrition app, as filled out by *current users*. The most important reasons were gaining insight into their own dietary pattern (113/276, 40.9%), losing weight (112/276, 40.6%), and maintaining body weight (96/276, 34.8%). More specific goals such as gaining insight into a specific meal moment (56/276, 20%), gaining insight into healthier alternatives for specific food products (50/276, 18%), or aiming to reduce snacking (48/276, 17%) seem less relevant.

Table 5 shows the most important reasons to stop using a nutrition app, according to *former users*. The following reasons were not selected in the survey and are therefore left out of the table: “App could not be personalized to my needs”; “Too many advertisements”; “It was difficult to keep track of personal progress”; and “It was unclear how to use the app.”

The most frequently mentioned reason was that using the app required too much time (14/38, 36.8%). Other frequently mentioned reasons were that the goal for which the app was installed was not important anymore (6/38, 15.8%) or that the app was not providing new information any longer (5/38, 13.2%). Remarkably, quitting the use because the database was not reliable (1/38, 3%) was mentioned by only 1 participant. The fact that the app could not be personalized to the users’ needs or that it was difficult to keep track of personal progress were both not mentioned. The same holds for too many advertisements in the app or that it is unclear how the app works.

Table 6 shows the most important reasons for not using a nutrition app, as answered by the group of *nonusers*. The most frequently mentioned reasons were no need to gain insight into dietary pattern (379/1106, 34%), followed by not seeing the need to use a nutrition app (360/1106, 30%), or having never heard of nutrition apps (235/1106, 21%). Moreover, in this group, the time investment seems to be a barrier because 16.6 (184/1106) of the participants mentioned not having time to use a nutrition app. Privacy does not seem to be an important barrier because being afraid that the data will not be treated confidentially was only mentioned by a relatively small part of the group (108/1106, 10%).

**Table 4:** Most important reasons to use a nutrition app in current users (n=276)^a^.

	Values, n (%)
Gaining insight into own dietary pattern	113 (40.9)
Losing weight	112 (40.6)
Maintaining body weight	96 (34.8)
Gaining insight into macronutrient intake, for example, protein	91 (33.0)
Aiming to eat healthier	85 (30.8)
Improving my health	85 (30.8)
Gaining insight into specific products and nutrients	78 (28.3)
Gaining insight into dietary pattern and physical activity	78 (28.3)
Gaining insight into a specific meal moment	56 (20.3)
Gaining insight into healthier alternatives for specific products	50 (18.1)
Aiming to reduce snacking	48 (17.4)
Other reasons	2 (0.8)

^a^Participants could indicate multiple reasons; therefore, percentages do not add up to 100%.

**Table 5:** Most important reasons to quit using a nutrition app in former users (n=38)^a^.

			Values, n (%)
It costs too much time			14 (36.8)
Goal for which I installed the app was not important anymore			6 (15.8)
The app was not providing new information anymore			5 (13.2)
I reached the goal for which I installed the app			3 (7.9)
App was not user-friendly			3 (7.9)
Too little or inappropriate feedback on dietary intake			2 (5.3)
Functionalities of the app were too limited			1 (2.6)
Database with food products was not reliable			1 (2.6)
**Other reasons**			5 (13.2)
	No space for it on my phone	1 (2.6)	
	The app triggered unhealthy/obsessed behaviors	2 (5.3)	
	I had to monitor physical exercise	1 (2.6)	
	I did not achieve the desired result	1 (2.6)	

^a^Participants could indicate multiple reasons; therefore, percentages do not add up to 100%.

**Table 6:** Most important reasons for not using a nutrition app in nonusers (n=1106)^a^.

			Values, n (%)
No need to gain insight in own dietary pattern			379 (34.2)
Do not see the point in using a nutrition app			329 (29.7)
Never heard of it			235 (21.2)
No time to use a nutrition app			184 (16.6)
Not involved in eating differently or healthier			165 (14.9)
Do not feel like learning how a nutrition app works			156 (14.1)
Afraid that data will not be treated confidentially			108 (9.7)
**Other reasons**			116 (8.2)
	User-unfriendliness or other obstacles	35 (2.5)	
	Sufficient knowledge on healthy nutrition	26 (1.8)	
	No need to use a nutrition app	31 (2.2)	
	Not possible owing to illness, age, or specific diet	11 (0.8)	
	Other	13 (0.9)	

^a^Participants could indicate multiple reasons; therefore, percentages do not add up to 100%.

## Discussion

### Principal Findings

In our study, we found that there are numerous functionalities in nutrition apps that contribute to consumers’ intentions to use and maintain using these tools. In total, 3 different phases of app use, 10 user-centric app aspects, and 46 associated app functionalities were identified. We found that consumers encounter several difficulties and barriers in using nutrition apps for a longer period. The qualitative study provided insights into the needs, perceptions, and opinions on app aspects that are important to consider in developing effective nutrition apps. Both by users and nonusers, these aspects were considered as important to include in nutrition apps, and no large differences between the groups were found. Our findings undermine the importance of a participatory approach when designing mHealth interventions, ensuring that the intervention addresses the target user’s needs and that the applied technology is easy to use to be successfully implemented in real life. This shows the relevance of not only evaluating the effectiveness of mHealth interventions by assessing health outcomes (eg, what does the use of a nutrition app do with nutritional behavior) but also by including user evaluations of various app aspects for effective implementation (eg, what is important for users to be engaged with nutrition apps) [31].

### Comparison With Prior Work and Recommendations

#### Overview

To the best of our knowledge, this study is the first to identify the different phases of nutrition app use, including user-centric app aspects and key app functionalities. We add to the literature by providing a complete overview of which app functionalities are important in each phase of app use, according to both users and nonusers. Several other studies have examined consumers’ preferences and barriers to using diet and nutrition apps. Here, the findings of these studies will be described and compared with our findings.

#### Findings Per Use Phase

In the starting phase, personalization of different nutrition app features seems to be a promising strategy for user engagement. App features should therefore be customizable and tailored to individual needs and goals, which is also described in the review by König et al [29]. Their findings are in accordance with our survey results, in which we found that especially personalization of the food tracking feature (eg, the possibility to track food intake at a convenient time) was important in the starting phase. Furthermore, we showed the importance of a clear purpose and introduction in the starting phase. A study by Dennison et al [32] evaluating mHealth app use found that participants want to be made fully aware of what the app can do before use. However, users are unlikely to read lengthy instructions and terms and conditions [32]. This emphasizes the importance of a clear and short introduction (eg, a tutorial) when setting up the app.

In the use phase, we found that user-friendliness and the food product database were among the most important app aspects. In a large web-based survey among European consumers, Vasiloglou et al [25] found that one of the primary criteria for selecting nutrition apps was ease of use. An app was less likely to be selected in case of issues with the food product database, such as incorrect nutritional information, a database that does not include local foods, or a database that omits major foods [25]. Several issues relating to the food database were mentioned in the FGDs, ranging from too detailed information and too many options for 1 product to questioning the reliability of the nutritional information provided. Issues with the food database can have consequences for nutrition app selection by consumers [25]. Other studies confirmed that the reliability and quality of the food database are common issues in nutrition apps and that the accuracy of nutrient information is sometimes questionable [33-36].

Although the current state of evidence supports that gamification can have a positive impact on changing health behaviors [37], including a gamification element in a nutrition app received one of the lowest ratings in our survey, meaning that consumers did not see the need to include this functionality in a nutrition app. Another functionality that received a low rating was interaction through social media. This was also found in the review by Snizay et al [38] on engagement with mHealth apps. They found that social support factors (social media and social competition) are not universally useful and might even cause disengagement by triggering negative emotions.

In the end phase, the ability to set new and achievable goals was one of the most important elements of the survey according to users. In the literature, achievable goal setting is identified as a promising facilitator for achieving behavior change [39]. Snizay et al [38] showed that goal setting was related to sustained engagement with mHealth and well-being apps. Incorporating a way to set daily and achievable goals, therefore, seems promising to keep users engaged in the final phase. In addition, nutrition apps should continuously offer new information, facts, and advice to keep users interested and engaged over a longer period.

The user-centric app aspects that arose from our qualitative study included several other validated BCTs such as monitoring and providing feedback. Broadly, the app elements that we identified are on the one hand elements that relate to these BCTs, such as factual information on nutrient intake (information), a visual progress overview (monitoring), and feedback messages (feedback), and on the other hand, the more technical design aspect of the app, such as a tutorial and a reliable database. Our study adds to the literature by showing that, for users, not only these technical aspects but also the way BCTs are implemented are important app elements. This suggests that the BCT mechanisms are not only considered as effective theoretical interventions to include in mHealth apps by health psychologists [13-16] but are, next to technical design aspects, also considered as critical elements from the user perspective.

Differentiating between the phases of use is a relevant approach to changing health behavior. There are several other theoretical models in the field of health behavior change that make a distinction between different stages, such as the Transtheoretical Model. This model describes stage-specific characteristics for behavior change and suggests that behavioral change is a dynamic process, comprising the precontemplation, contemplation, preparation, action, and maintenance stages [40]. Our 3 phases of nutrition app use overlap with the preparation (start), action (use), and maintenance (continuation) stages of the Transtheoretical Model. A user typically goes through the start phase, the use phase, and the end phase, where they reach a point where the app will be used permanently or occasionally or will not be used anymore. The decision for continued use is interlinked with the aspects mentioned in the earlier phases of use. The precontemplation and contemplation phases are lacking in our research. In these phases, awareness and intention are created to start performing a certain behavior. Because in our study participants were required to use a nutrition app as a home-use test and did not start using the app out of their own intrinsic motivation, we cannot make any statements about what app elements are important to create the intention to start using the app. Our starting phase starts when the app is installed and a user starts to navigate through the app, but obviously, in practice, this is preceded by an “intention to start” phase. The Unified Theory of Acceptance and Use of Technology sheds some light on this intention phase and distinguishes 4 factors that are important in this phase: performance expectancy (eg, the belief that a nutrition app will help), effort expectancy (eg, the expectancy that the app will be easy to use), social influence (eg, the beliefs of others who are important), and facilitating conditions (eg, the degree to which a user believes that an infrastructure exists to support use of the system) [41]. Future research is needed to obtain a better understanding of app requirements that are crucial to get users to install and open the app in the first place.

#### Quit or Start Nutrition App Use

The high time investment that consumers perceived as one of the main barriers was also one of the main reasons for quitting nutrition app use in the survey. The fact that using a nutrition app is perceived as time-consuming can be a result of several issues, such as poor user-friendliness or bad quality of the food product database. Poor user-friendliness is indeed a common issue for nutrition apps [29]. According to a study by Zečević et al [26], technical issues can also be a barrier in this regard. Therefore, nutrition app developers should pay particular attention to a complete and trustworthy food database and the user-friendliness of the app.

Finally, in our qualitative study, we found that nutrition apps can be specifically helpful for new users and that the learning curve is steepest in the beginning. A recent study by Samoggia et al [42] shows that a nutrition-information app is indeed mostly effective among consumers with limited knowledge. One of the main reasons to use a nutrition app that emerged from our survey was to gain more insight into one’s dietary pattern. Lowe et al [43] also suggest that nutrition app use can help increase nutrition knowledge and awareness of consumption practices. Losing and maintaining body weight were other frequently mentioned reasons to use a nutrition app. This is in line with the findings of the review by König et al [29]: the main goals of using nutrition apps were related to food tracking, diet improvement, and weight management. Several review studies show the effectiveness of nutrition apps in reducing body weight in different consumer groups [7,44,45]. Therefore, given their magnitude, low cost, and easy accessibility, nutrition apps are promising tools for consumers with limited knowledge to adopt healthier eating behaviors and to lose or maintain body weight.

### Limitations and Future Research

Our study has some limitations that need to be addressed. First, in both studies, a relatively large number of higher-educated consumers and, in the qualitative study, consumers interested in nutrition apps participated, which might have biased our results. However, these groups are probably also the ones that make use of nutrition apps in real life; therefore, we expect that the results might in fact be quite representative for implementation in real life. mHealth apps seem to be mostly designed to help a group of higher-educated and motivated consumers, which can be considered a limitation of these types of tools. This means that for unmotivated or lower-educated consumers, other types of interventions might be more suitable, which should be tested in future research. In this qualitative study, free versions of the 6 nutrition apps were included. It might be possible that the paid upgrades of the apps included better or additional features. Furthermore, the consumers who participated in the FGDs had no clear goal when using the app during the home-use test because we asked them to install the app for our study. The fact that they had no clear personal goal with the app, combined with the poor user-friendliness of some of the apps, might have caused early discontinuation by some users. In the survey, all 46 nutrition app functionalities were assessed on 7-point Likert scales and rated on importance. The rating of these 46 functionalities might have caused fatigue; however, we did include motivational messages in between questions, and respondents with no variability in their answers were removed from the analyses. The top 3 most important app functionalities per use phase were based on mean scores. This approach was chosen because we aimed to validate all user-centric app aspects and functionalities that we found in the FGDs. Including all of them in a ranking task or choice-based experiment was not feasible. This method may have caused consumers to rate almost all aspects as important, as they were not forced to make a choice. This made it difficult to draw conclusions on which specific elements are most important to include in nutrition apps. Therefore, a choice-based experiment with a selection of nutrition app functionalities is recommended, making it possible to uncover the trade-offs between different app functionalities. Another limitation is that we examined consumers’ intentions and preferences, and we did not study the effect of nutrition app functionalities on actual dietary behavior. Such an intervention would be recommended for future research.

Finally, in both studies, we mainly focused on the elements and functionalities in the nutrition app itself. However, user characteristics and characteristics of the eating context could also influence consumers’ intentions to use nutrition apps. The framework of König et al [29] highlights that besides technological reasons, the characteristics of the potential user, the interplay between user and technology, and the social environment also impact whether a nutrition app is used. In addition, Flaherty et al [46] stress the increasing importance of situational involvement and individual characteristics in engagement with mHealth apps.

### Conclusions

Nutrition apps should be supportive in all 3 phases of use (start, use, and end) to increase consumers’ intentions to start and maintain the use of these apps and achieve a change in dietary behavior. In the starting phase, a clear purpose, introduction, and personalization are important functionalities. In the use phase, a high-quality, credible food product database and user-friendliness are particularly important. In the end phase, the app should continuously offer new information and the possibility of setting new personal goals. High time investment is an important reason to quit the use of a nutrition app. Several other issues with nutrition apps (ie, poor user-friendliness and not offering new information anymore) need to be addressed first before long-term use can be achieved. Because almost all app functionalities in our study were considered as important by both users and nonusers, a choice-based experiment with different nutrition apps is recommended as a next step.

## Appendix

Multimedia Appendix 1 Supplementary tables and figures.

## Abbreviations

BCT: behavior change technique
FGD: focus group discussion
mHealth: mobile health
