# Supplementary material for: Incorporating Consumers’ Needs in Nutrition Apps to Promote and Maintain Use: Mixed Methods Study
Source: JMIR Mhealth Uhealth. 2023 Jun 20;11:e39515. doi: 10.2196/39515 (PMC10337335; doi:10.2196/39515)
Supplement: Multimedia Appendix 1 [file mhealth_v11i1e39515_app1.docx]

**Table S1.** Key functionalities of nutrition apps per user-centric app aspect

| **Phase 1: Start** | | | **Phase 2: Use** | | | | | | **Phase 3: End** |
| --- | --- | --- | --- | --- | --- | --- | --- | --- | --- |
| **User-centric app aspects** | | | | | | | | | |
| **1.**  **Purpose** | **2.**  **Introduction** | **3.**  **Personalization** | **4.**  **User-friendliness** | **5.**  **Database** | **6.**  **Information** | **7.**  **Monitoring** | **8.**  **Feedback** | **9.**  **Communication** | **10.**  **Continuous engagement** |
| The app has a clear purpose | Short tutorial is available | Possibility to set personal goals | Easy navigation through the app | Clear definition of product categories | Factual information on food is provided: calories, nutrients, ingredients | Visual overview of progress in graphs, lines, statistics | Positive feedback is provided | Possibility to connect with others through forums, social media or coach | Possibility to set realistic and achievable goals |
| Description of key app functions | Immediately clear how app should be used | Possibility to set personal nutrition knowledge level | Pleasant look and feel | Logical product categories | Healthier product alternatives are offered | Ideas, suggestions and recipes are offered (on demand) | Variation in feedback messages (topic and style) | Tips, ideas and recipes are offered | Possibility to set new personal goals |
| Variety of functions (nutrition, exercise, sport, meditation etc.) |  | Possibility to track food intake at own time | Functional, engaging animations and imaging | Complete and reliable food database | Information that matches personal knowledge level | Friendly, adjustable notifications to fill in daily intake | Healthy choices are encouraged without pressure or playing on guilt | Clear privacy statements | New and relevant information is continuously offered |
|  |  | Detailed entry of personal information on physical activity and lifestyle | Quick and easy entry of food products | Scientific and credible endorser of database | Awareness of own behavior is created | Reminder when personal goals are not achieved | Personal saving or reward system (virtual or real) |  | Possibility to adjust detail level of personal information |
|  |  | Possibility to prioritize certain functions | Integration with other smart devices | Database with country-specific products |  |  | Gamification element |  | Possibility to make app dormant and set periodic reminders to start usage again |
|  |  | Possibility to create personal lists and meals | Consistent use of language, preferably Dutch |  |  |  |  |  |  |
|  |  | Possibility to social media usage and notifications | Choice in measuring units |  |  |  |  |  |  |
|  |  |  | Limited advertisements in free version |  |  |  |  |  |  |

**Table S2.** Ratings per app functionality with corresponding phase and user-centric-app-aspect

|  | **App functionality** | **Phase** | **User-centric app aspect** | **Mean rating + SD** |
| --- | --- | --- | --- | --- |
| 1. | Complete and reliable product database | Use | Database | 5.58 ± 1.41 |
| 2. | Easy navigation through the app | Use | User-friendliness | 5.56 ± 1.36 |
| 3. | Limited advertisements in free version | Use | User-friendliness | 5.53 ± 1.51 |
| 4. | Scientific and credible endorser of database | Use | Database | 5.52 ± 1.38 |
| 5. | Quick and easy entry of food products | Use | User-friendliness | 5.50 ± 1.39 |
| 6. | Consistent use of language, preferably Dutch | Use | User-friendliness | 5.46 ± 1.50 |
| 7. | Clear privacy statements | Use | Communication | 5.46 ± 1.51 |
| 8. | Immediately clear how app should be used | Start | Introduction | 5.45 ± 1.33 |
| 9. | The app has a clear purpose | Start | Purpose | 5.40 ± 1.37 |
| 10. | Possibility to track food intake at own time | Start | Personalization | 5.33 ± 1.45 |
| 11. | Logical, user centric product categories | Use | Database | 5.32 ± 1.36 |
| 12. | Clear definition of product categories | Use | Database | 5.30 ± 1.37 |
| 13. | Database with country-specific products | Use | Database | 5.28 ± 1.50 |
| 14. | Healthier product alternatives are offered | Use | Information | 5.23 ± 1.42 |
| 15. | Possibility to set realistic and achievable goals | End | Motivational aspects | 5.23 ± 1.44 |
| 16. | Choice in measuring units | Use | User-friendliness | 5.19 ± 1.44 |
| 17. | Possibility to set personal goals | Start | Personalization | 5.18 ± 1.43 |
| 18. | Possibility to create personal lists and meals | Start | Personalization | 5.15 ± 1.36 |
| 19. | Possibility to set new personal goals | End | Motivational aspects | 5.13 ± 1.45 |
| 20. | Factual information on food is provided: calories, nutrients, ingredients | Use | Information | 5.13 ± 1.46 |
| 21. | Description of key app functions | Start | Purpose | 5.13 ± 1.38 |
| 22. | Pleasant look and feel | Use | User-friendliness | 5.05 ± 1.46 |
| 23. | Healthy choices are encouraged without pressure or playing on guilt | Use | Feedback | 5.01 ± 1.51 |
| 24. | Short tutorial is available | Start | Introduction | 5.00 ± 1.45 |
| 25. | Visual overview of progress in graphs, lines and statistics | Use | Monitoring | 4.98 ± 1.49 |
| 26. | Awareness of own behavior is created | Use | Information | 4.98 ± 1.43 |
| 27. | Variety of functions (nutrition, exercise, sport, meditation etc.) | Start | Purpose | 4.90 ± 1.49 |
| 28. | Detailed entry of personal information on physical activity and lifestyle | Start | Personalization | 4.89 ± 1.43 |
| 29. | New and relevant information is continuously offered | End | Motivational aspects | 4.88 ± 1.44 |
| 30. | Functional, engaging animations and imaging | Use | User-friendliness | 4.88 ± 1.51 |
| 31. | Information that matches personal knowledge level | Use | Information | 4.88 ± 1.48 |
| 32. | Ideas, suggestions and recipes are offered on demand | Use | Monitoring | 4.87 ± 1.47 |
| 33. | Possibility to prioritize certain functions | Start | Personalization | 4.86 ± 1.37 |
| 34. | Possibility to adjust detail level of personal information | End | Motivational aspects | 4.82 ± 1.47 |
| 35. | Possibility to set personal nutrition knowledge level | Start | Personalization | 4.81 ± 1.47 |
| 36. | Tips, ideas, blogs and recipes are offered | Use | Communication | 4.67 ± 1.57 |
| 37. | Positive feedback is provided | Use | Feedback | 4.66 ± 1.57 |
| 38. | Possibility to make app dormant and set periodic reminders to start usage again | End | Motivational aspects | 4.64 ± 1.57 |
| 39. | Reminder when personal goals are not achieved | Use | Monitoring | 4.61 ± 1.51 |
| 40. | Friendly, adjustable, notifications to fill in daily intake | Use | Monitoring | 4.52 ± 1.51 |
| 41. | Variation in feedback messages (topic and style) | Use | Feedback | 4.47 ± 1.50 |
| 42. | Personal saving or reward system (virtual or real) | Use | Feedback | 4.26 ± 1.82 |
| 43. | Integration with other smart devices | Use | User-friendliness | 4.08 ± 1.78 |
| 44. | Possibility to set social media usage and notifications | Start | Personalization | 3.80 ± 1.90 |
| 45. | Gamification element | Use | Feedback | 3.76 ± 1.79 |
| 46. | Possibility to connect with others through forums, social media or coach | Use | Communication | 3.54 ± 1.81 |
